# Supplementary material for: Carbon Dots Boost dsRNA Delivery in Plants and Increase Local and Systemic siRNA Production
Source: Int J Mol Sci. 2022 May 10;23(10):5338. doi: 10.3390/ijms23105338 (PMC9141514; doi:10.3390/ijms23105338)
Supplement: Supplementary file 1 [file ijms-23-05338-s001.zip › ijms-1694753-supplementary.pdf]

SUPPLEMENTARY MATERIAL

Table S1. Atomic concentration table (%) for the sCD and gCD carbon dots according to the XPS analysis.

**sCD**	**C1s**	**N1s**	**O1s**
RSF	0.31	0.50	0.73
Corrected RSF	6.27	10.18	15.32
Atomic concentration	74.36	16.32	9.32
**gCD**	C1s	N1s	O1s
RSF	0.31	0.50	0.73
Corrected RSF	6.27	10.18	15.32
Atomic concentration	73.99	16.70	9.32

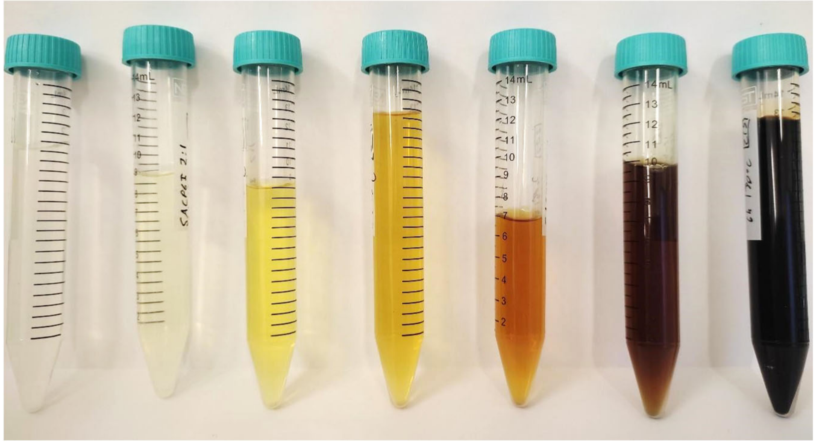

Figure S1. Serial dilutions (1:5) of the saccharose carbon dots (sCD). The aspect of the glucose carbon dots (gCD) dilutions resulted similar.

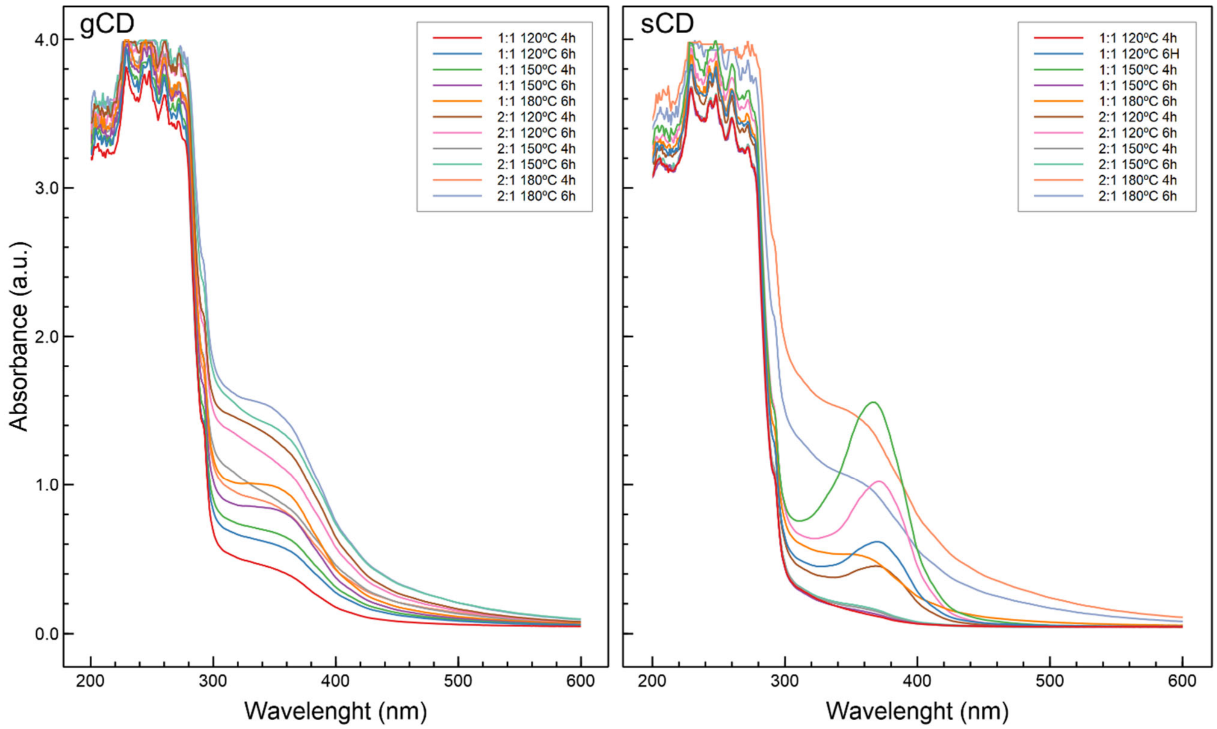

Figure S2. Absorption spectra of carbon dots prepared with different carbon precursor (glucose or saccharose) according to the proportion (weight:weight) between the carbon precursor and the bPEI (2 KDa), the temperature (ºC) and time of reaction (4-6 h) in the hydrothermal synthesis.

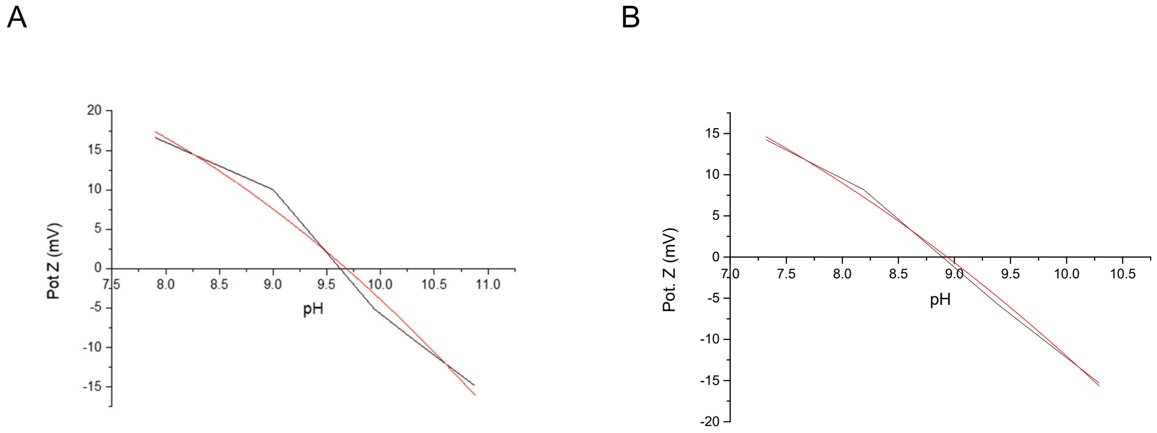

Figure S3. Determination of isoelectric points of the carbon dots obtained from glucose, gCD (A) and saccharose, sCD (B) passivated with the bPEI of 2 kDa as obtained using 180 ºC of reaction temperature for 6 h. The CDs were dialyzed with the 1 KDa exclusion membrane.

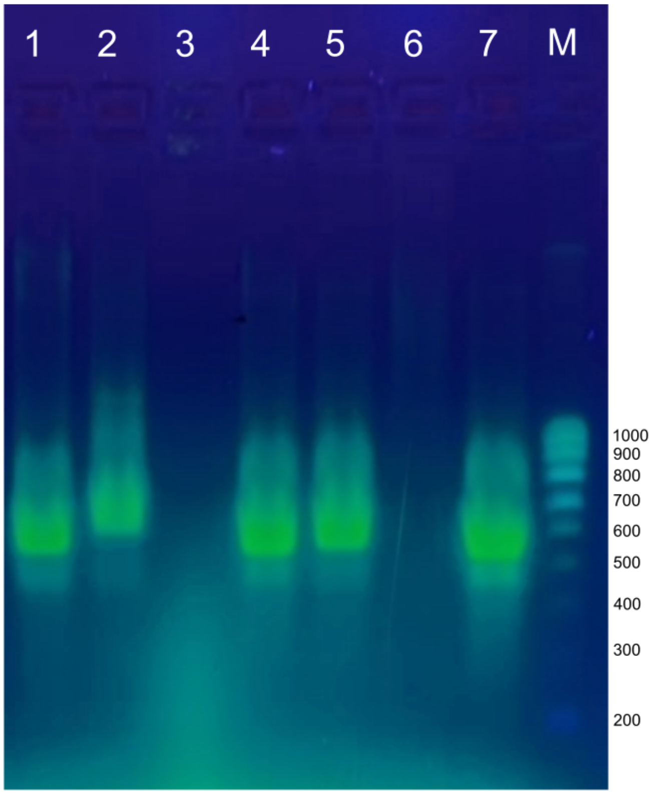

Figure S4. Migration in 2% agarose gel of FITC-labeled dsRNA when naked or coated with the CDs in different proportions weight:weight. (1) gCD:dsRNA (1:10); (2) gCD:dsRNA (1:5); (3) gCD; (4) sCD:dsRNA (1:10); (5) sCD:dsRNA (1:5); (6) sCD; (7) dsRNA; (M) Molecular weight marker: NZY Tech Ladder V. CDs without dsRNA are out of the image as they have migrated to the negative pole (top).

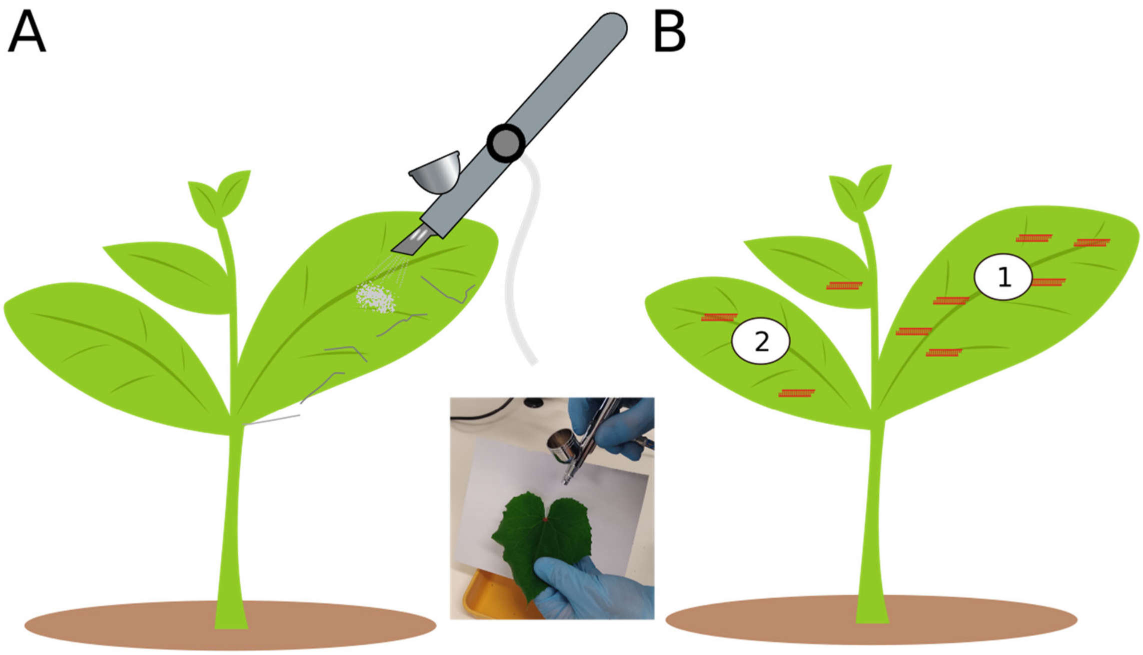

Figure S5. DsRNA spraying and sampling on cucumber leaves. Naked dsRNAs or gCD-dsRNAs were applied by spraying with the airbrush onto the cucumber leaves, while avoiding the spraying to other parts of the plants with a foil (inlet image). Samples for the RNA extractions and the quantitations were taken at points 1 (local leaf) and, at point 2 (distal leaf), for investigating the systemic movement of the sprayed dsRNA. The samplings were made at three days post application for detecting the dsRNAs and the vsiRNAs. Prior the sampling for the RNA extractions the leaves were strongly washed with deionized water.
